# Secure Trust-Based Blockchain Architecture to Prevent Attacks in VANET

**DOI:** 10.3390/s19224954

**Published:** 2019-11-14

**Authors:** Adnan Shahid Khan, Kuhanraj Balan, Yasir Javed, Seleviawati Tarmizi, Johari Abdullah

**Affiliations:** 1 Faculty of Computer Science and Information Technology, Universiti Malaysia Sarawak, Kota Samarahan 94300, Malaysia; 17020126@siswa.unimas.my (K.B.); yjaved@psu.edu.sa (Y.J.); swati@unimas.my (S.T.); ajohari@unimas.my (J.A.); 2 Department of Computer Science, Prince Sultan University, Riyadh 11586, Saudi Arabia

**Keywords:** VANET, trust model, blockchain, architecture, privacy, authentication, security

## Abstract

Vehicular ad hoc networks (VANET) are also known as intelligent transportation systems. VANET ensures timely and accurate communications between vehicle to vehicle (V2V) and vehicle to infrastructure (V2I) to improve road safety and enhance the efficiency of traffic flow. Due to its open wireless boundary and high mobility, VANET is vulnerable to malicious nodes that could gain access into the network and carry out serious medium access control (MAC) layer threats, such as denial of service (DoS) attacks, data modification attacks, impersonation attacks, Sybil attacks, and replay attacks. This could affect the network security and privacy, causing harm to the information exchange within the network by genuine nodes and increase fatal impacts on the road. Therefore, a novel secure trust-based architecture that utilizes blockchain technology has been proposed to increase security and privacy to mitigate the aforementioned MAC layer attacks. A series of experiment has been conducted using the Veins simulation tool to assess the performance of the proposed solution in the terms of packet delivery ratio (PDR), end-to-end delay, packet loss, transmission overhead, and computational cost.

## 1. Introduction

**Figure 1:**
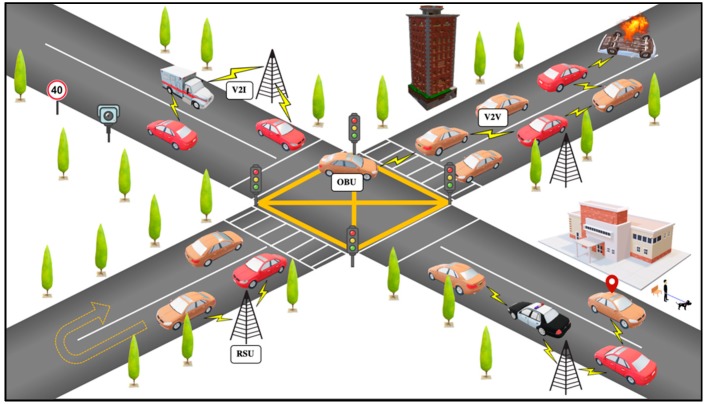
Basic vehicle ad hoc networks (VANET) communication.

Vehicle ad hoc networks (VANET) emerged as a subset of a mobile ad hoc network (MANET) [1] application. VANET is considered a substantial approach for intelligent transportation systems (ITS) [2]. VANET has recently been the focus of various researchers in the wireless mobile communication field. The aim of VANET is to provide inter-vehicle communication and roadside units to vehicle communication to increase road safety and improve local traffic flow and the efficiency of road traffic by providing accurate and timely information to road users [3]. In VANET, vehicles are used as network nodes, as seen in Figure 1.

There are two types of communications in VANET, which are vehicle to vehicle (V2V) and vehicle to infrastructure (V2I) communications [4]. The on-board units (OBUs) and road side units (RSUs) in VANET establish a connection among themselves with the help of dedicated short-range communication (DSRC) in a single or multi-hop communication [5,6,7].

VANET offers various services and applications to the users, most of which are concerned with the safety of the drivers, infotainment, and navigational aid [8]. There are two types of information shared in VANET: safety (vehicle speed warning, curve warning) and non-safety information (value-added comfort application) [9]. By default, safety information is given a higher priority in VANET as compared to non-safety information, since safety information notifies drivers of expected dangers to allow an early response [10,11]. Despite the benefits offered by VANET, it comes with challenges, especially in terms of the security and privacy of users and transmitted messages [12].

As vehicles enter and exit highways, they require certain safety information, such as traffic congestion and road conditions, to make decisions on which route to take to their destination. It is essential that this information be delivered in a timely manner; otherwise, it could lead to delays in reaching the destination safely [13]. In certain scenarios, some malicious nodes refuse to relay or even intentionally modify the required safety messages before transmitting to the requesting user, which could result in longer delay or fatalities. Besides this, characteristics of VANET (e.g., high mobility, volatility) which are distinct from other wireless communication networks have caused VANET to be susceptible to numerous internal and external attacks [14]. Due to the decentralized structure and dynamic topology of VANET, the security of the vehicles, users, and data has become essential, since the identification of malicious or faulty nodes or users has become difficult [7]. In VANET, vehicles exchange sensitive information and traffic changes with each other [15]. However, a lack of authentication of this information can result in malevolent attacks which present harm to drivers [16]. However, for messages to be authenticated, the vehicles in the network are tracked for their identification and whereabouts at any given time [17], compromising the privacy of the users. Hence, there must be a perfect balance between authentication and preserving the privacy of the users [18]. Several researchers have developed various techniques for preserving privacy, such as pseudonyms [19] and anonymous authentication [20], which could achieve the goal of preserving the privacy of the users, as long as the pseudonyms cannot be linked to the user. Nevertheless, these schemes may not be very secure, because reported traffic information can be utilized to link the pseudonyms to the users, as vehicles do not change their pseudonyms during information exchange [21]. Not only that, the availability of abundant valid pseudonyms for each vehicle makes VANET vulnerable to attacks, such as Sybil attacks, as the pseudonyms could be used to correctly authenticate non-existing vehicles [22]. Although solutions that can provide secure communication channels against external attacks are available, trust management and privacy protection for vehicles are still open issues for VANETs [23]. Therefore, designing a secure VANET demands that three key elements be considered—security, privacy, and trust [24]—to reduce or prevent any attacks in the network. This paper is organized as follows. Section 2 presents the related work. Section 3 explains the motivation of the proposed approach. In Section 4, the proposed blockchain in VANET is presented and the simulation environment is set-up in Section 5, followed by the performance and security analysis of the proposed solution under results and discussion in Section 6. Finally, Section 7 concludes the paper, along with future work.

## 2. Related Works

There have been numerous security schemes proposed by various researchers to address the security and privacy issues in VANETs. This section highlights some of the existing approaches that focused on similar problems in VANET using similar techniques to the proposed solution. An anonymous and lightweight authentication scheme smart card (ASC) was proposed by Ying to address privacy-preserving problems, such as legitimacy of users and messages transmitted across the network [25]. The authentication of users and messages are done using low-cost cryptographic operations. This protocol does not only verify the identity of the users and authenticate messages communicated, but it also guarantees anonymity of users. Anonymity in this scheme is achieved by dynamically generating login identities for users using smart cards to replace users’ actual identity, thereby hiding the real identities of users from attackers. The dynamic login identity is updated periodically to provide anonymous authentication. The researchers also proposed a dynamic password change without depending on a trusted authority (TA), so that the scheme is resistant to various attacks such as impersonation and offline password guessing attacks. The messages in ASC are authenticated using two hash chains to minimize computation complexity. Apart from that, messages are appended with timestamps obtained from GPS devices to validate the freshness of the messages and minimize replay attacks. The performance of ASC was evaluated using VanetMobiSim in terms of communication and computation overheads, end-to-end delay, and average packet loss ratio. From the simulation, ASC was found to perform better and have better efficiency than other baseline protocols. Nevertheless, a major drawback of ASC is that the frequent update of login identity and user password will introduce higher computation cost in the network. Not only that, the frequent updating of login identity may open new opportunities for attackers to launch attacks such as impersonation or identity theft, since the login identity changes periodically, resulting in increased difficulty in detecting malicious nodes.

Wazid proposed a decentralized lightweight authentication and key agreement protocol (LAKAP) for VANETs, which uses one-way hash functions and bitwise exclusive OR (XOR) operations [26]. The proposed lightweight protocol highlights some features: it allows dynamic road side unit addition in the network after initial deployment, provides RSU to RSU key establishment, and has anonymity and intrackability, among other additional features. Apart from that, the solution exhibits three authentication approaches: between vehicles, between vehicle and its respective cluster heads (CHs), and between CHs and their RSUs. The researchers adopted a cluster-based network model to reduce the computation and communication overheads. In this proposed protocol, the CHs are elected based on trust values and strong connectivity. Only vehicles with high trust values and strong connectivity are selected as the CHs. Each of three authentication approaches also establishes a keypair for the nodes involved. Besides this, a key is also established between neighboring RSUs to maintain secure communication using the keys. The proposed lightweight authentication scheme uses timestamps to prevent replay attacks in the network. The passwords of each vehicles are updated periodically to improve the security of the user and information. Using a computer running on Intel Xeon E5-1620 v2 processor with 16 GB RAM provided by the Faculty of Computer Science and Information Technology, Universiti Malaysia Sarawak, the performance of the proposed scheme was evaluated using Network Simulator 2.35 (NS-2.35) in terms of communication and computation overheads, throughput, end-to-end delay, and packet delivery ratio (PDR). From the analysis, the lightweight authentication and key agreement protocol was found to encounter low communication and computation costs. However, in a high-mobility and dynamic environment, it is difficult to select vehicles as CHs due to the high velocity of the vehicles. This could lead to inefficiency as well as unnecessary waste of energy of the nodes. Not only that, the periodic update of vehicles’ passwords could also lead to an increased computational overhead in the network, as the OBUs need to compute the identity and password of each vehicle before a new password could be passed to the vehicle.

Rajput proposed a hybrid approach for privacy-preserving authentication scheme (HEPPA), which combines features of pseudonym-based approaches and group signature-based approaches, with conditional anonymity [27]. According to the researchers, the real identity of an attacker can be uncovered during the detection of a malicious activity. This hybrid approach uses a simple and lightweight pseudonym which provides conditional anonymity. The pseudonym provides a trapdoor mechanism to enable the detection of malicious nodes and subsequently revoke such users from the network. They also introduced a cloud-assisted modular architecture that acts as a certification authority (CA), which is responsible for vehicle registration and verification of their credentials. Another feature of the hybrid approach is the grouping of vehicles based on regions that are managed by the CA using similar credentials, so that attackers cannot differentiate between vehicles in the group. The scheme does not require a vehicle to manage the certificate revocation list (CRL) to reduce processing overhead on OBU and bandwidth consumption. The researchers used elliptic curve cryptography (ECC) as the cryptographic tool, in which the elliptic curve integrated encryption scheme (ECIES) is used for encryption and elliptic curve digital signature algorithm (ECDSA) is used for the signatures of the vehicles. The performance of the proposed hybrid approach was analyzed via simulation using vehicles in network simulation (Veins), in terms of communication and computation overheads, end-to-end delay, PDR, and packet loss. The simulation results showed that the proposed hybrid approach is feasible enough to enhance the privacy of users in VANET. Nevertheless, a drawback of this approach is the region-based grouping of vehicles in the highly dynamic and high-speed network. Vehicles enter and leave a region quickly due to their high velocity, therefore increasing the difficulty in managing the groups by the CA.

Tangade proposed an efficient, scalable, and privacy-preserving authentication (ESPA) protocol using a hybrid cryptography approach for inter vehicle communications [28]. ESPA is comprised of two phases—Phase I: V2I pre-authentication, and Phase II: V2V authentication. After off-line registration of vehicles and RSUs, the beacon signal of each vehicle is pre-authenticated by RSU during V2I communication to verify whether the vehicle belongs to any base stations in the network or is an unauthorized vehicle. The pre-authentication is carried out using asymmetric public key infrastructure (PKI). In the second phase, only vehicles that have been pre-authenticated can get involved in the V2V communication. ESPA considers V2V communication covered by the same agents of trusted authority, with vehicles having the same secure keys. ESPA was evaluated via simulation using NS-3.23, as well as traffic simulators, simulation of urban mobility (SUMO), and mobility model generator for vehicular network (MOVE), considering a two-lane, two-way highway. From the simulation analysis, ESPA was found to reduce transmission and computation overheads. According to the researchers, ESPA provides better security and meets the privacy properties requirements in VANET.

Cui proposed a secure privacy-preserving authentication scheme for VANET with cuckoo filter (SPACF) to enhance the security and privacy of users, as well as to minimize the communication overhead [29]. The researchers proposed to utilize the cuckoo filter and binary search methods to achieve a higher success rate than other PKI-based and identity-based authentication systems in the verification phase. Additionally, the researchers also proposed a new authentication scheme without bilinear pairings, which can result in a heavy computational cost. Cuckoo filter is a data structure that provides better search accuracy and search time and uses hashing functions. Every time a vehicle moves to a new RSU, it authenticates itself using the TA, which will then pass the vehicle’s information to verify the identity of the said vehicle. For every message distribution, a vehicle needs to generate a pseudo identity and corresponding signing key to increase the difficulty of attackers tracing the real identity of the users. Furthermore, RSUs use a batch verification approach to verify a set of messages without the bilinear pairings. Apart from that, SPACF also allows for group key generation when vehicles want to form a group, as well as group message signing and verification to allow group members to verify the signature of other members without the aid of RSU. The performance of SPACF was analyzed using a simulation software that was based on C++ in terms of communication, computation, and transmission overheads. From the analysis, SPACF has lower communication, computation, and transmission costs when compared to other security protocols.

The existing approaches discussed were selected as the benchmark protocols for this study as these approaches focus on enhancing security and preserving the privacy of users in the network. It can be seen that the existing approaches primarily focused on privacy-preserving and authentication schemes. However, other security requirements of VANET, such as availability, integrity, and non-repudiation, were not given much attention. This gives a gap for further improvement in VANET security with the consideration of implementing a much newer security-based technology that is out there in the current world. Thus, the solution proposed in this study attempts to improve the security of VANET by implementing a much up-to-date technology that could address the security requirements as a whole.

## 3. Motivation

This section discusses the challenges faced in the VANET environment and the implications they bring to the genuine nodes in the network during the occurrence of network attacks. The major challenge faced in the VANET environment is the mobility of the nodes, which can be an RSU, vehicles that are stuck in the traffic congestion, or one vehicle moving at a fast pace. The nodes within the system itself have challenges in communicating [6]. The mutual communication window during a high-velocity scenario is kept small, only a few seconds, due to the small transmission range. Besides, the communication system has to cope with the Doppler effect, frequent link failures, wastage of network bandwidth, and high end-to-end delay for high relative velocity [30]. Although nodes have a high period of message exchange, they must deal with the problems related to high vehicular traffic density, such as frequent data collision, channel fading, message dropping due to expired waiting time, and other interference problems. Due to the coverage area of vehicles, the connectivity can be lost with the high mobility and might travel in the opposite direction which makes the nodes’ connectivity extremely ephemeral. Long live context is lacking in VANET systems, where a hotspot with a long-life password from the user node is required and is at the same time impractical for securing volatile connections. Privacy in keeping their personal details private, protected, and not exposed by the drivers will be quite difficult when the system is set to give identities to all the individual vehicles in order to avoid Sybil attacks. Each vehicular node has the right to keep its personal details from other nodes while not violating the privacy, no matter of the situation. For example, during an accident, the legal investigation is allowed to access data without any denial, and it is an advantage for the liability that provides the opportunity for open investigation platform. 

Since there is no global authority that handles the standards for VANET systems, and the scale of the network increases every single day—approximately more than 750 million nodes—the problem of standardization arises. For instance, the standards for the same car brand are different in different countries or continents, and also totally distinguishable for different brands of car. A node in the context of the VANET system can be placed in a high-density network, such as a traffic jam, or in a low-density network, such as a highway roadway with no or fewer surrounding vehicles. In a low-density network, an advanced information message dissemination using a store-and-forward message is transmitted instead of the immediate message forwarding [31]. The same message has the possibility of repeating multiple times by the same vehicle due to this case scenario. In the case of a high-density network, this is achieved by choosing only selected vehicles given permission to send a repeated message. The density of the node is influenced by the road and time, which is commonly measured as high during the daytime compared to other times [32]. In the case of high density, the opposite must be achieved, with only selected vehicles allowed to send repeated messages. The vehicular node follows a predefined path instead of a random path, as different roads come with various characteristics [33]. Compared to rural and highway roads, urban roads are usually a dense roadway with numerous vehicles, buildings, and other obstacles. As the characteristics of the roadways differ, the movement pattern of the vehicular nodes varies too due to the challenge they pose for an efficient communication [6]. For example, urban roads are not as highly ordered as the highway roads in terms of movement. Heterogeneity is also a challenge in the VANET system, as every single node has its own characteristics depending upon its applications, be it either a stationary node, such as RSU, or a mobile node, such as the vehicles. Moreover, the nodes can be categorized into different levels according to the requirements of the application [34]. To support the heterogeneity characteristics, each vehicular node can be further classified into private, authority, and maintenance based on the vehicle, whereas RSU can be classified into able to emit data and completeness of ad-hoc features [6]. RSUs do not require a privacy feature, unlike the vehicular nodes. Therefore, the VANET system shall be able to provide services based on the requirements of singular nodes.

## 4. Proposed Blockchain in VANET

Many academicians and researchers are drawn to blockchain technology for its enormous benefits to be gained in vast fields, including academics, finance, medicine, and banking. To be precise, blockchain is a technology that is technically comprised of an unlimited number of blocks that are connected in a sequential order to form a blockchain. As this technology is potentially beneficial for expertise in vast fields, it has also gained the interest of many in resolving critical information dissemination issues in VANETs. Bitcoin cryptocurrency is the underlying support of blockchain technology that emerges from the decentralization and distribution of a computing paradigm that has the ability to provide privacy and security in peer-to-peer (P2P) networks [35]. In the VANET environment, this technology is a vital part that helps in managing the ground truth of information for automobiles due to the fact that any automobiles in the system can access the past event lists and its information if it is placed in the public blockchain.

The proposal is to generate a scheme whereby the trustworthiness of node and message passing in VANET is guaranteed by placing them in a public blockchain to act as a ground truth for other automobiles. The application of an existing blockchain to the VANET system is not sufficient, as event messages as a transaction form are adopted instead of the bitcoin transaction for the cryptocurrency feature. The reason for the variation made to the transaction is to ensure the suitability of features for the VANET system as an assurance of providing security for critical information dissemination and resolving the VANET issues. The variation method adapted adds new blocks based on event messages, similar to transactions in bitcoin, apart from the hashing sequences of blocks to be connected in chronological order to the blockchain. The scalability and timeliness of message dissemination is ensured in this system by implementing a local blockchain with independent chains from different geographical regions. A public blockchain is considered to store and manage all the node and message trustworthiness information given in a geographical region. Based on the type of blockchain, which could be either public or private, a different set of blockchain consensus mechanisms are offered. Therefore, the security and scalability level of the blockchain also depends on the vital role played by the consensus mechanisms. For simplicity, Table 1 shows all the notations, with their descriptions, used throughout the paper.

**Figure 2:**
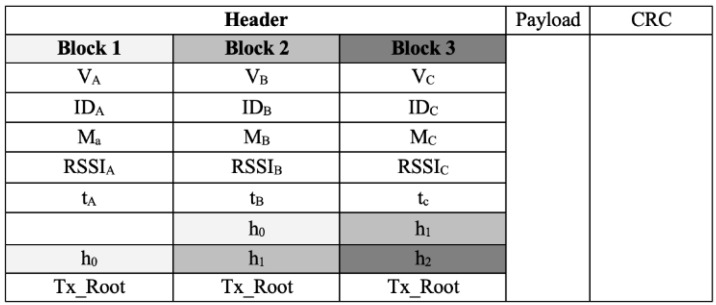
Proposed packet structure with blockchain integration.

A simple blockchain would not be suitable for the VANET issue discussed in this study. Hence, an improvised type of blockchain mechanism with some feature adaptation is proposed as a solution. Figure 2 depicts the improvised packet structure with the integration of a blockchain that was used for a secure communication by VANET components. Every block is comprised of vehicle *i* (V*_i_*), identity of vehicle *i* (ID*_i_*), message *i* from vehicle *i* (M*_i_*), relative signal strength indicator value of vehicle *i* (RSSI*_i_*), timestamp of vehicle *i* (t*_i_*), hash value, and transaction root value. For event messages, the safety event messages are used herein where the blockchain is the medium of trustworthiness for the event messages in VANET.

**Figure 3:**
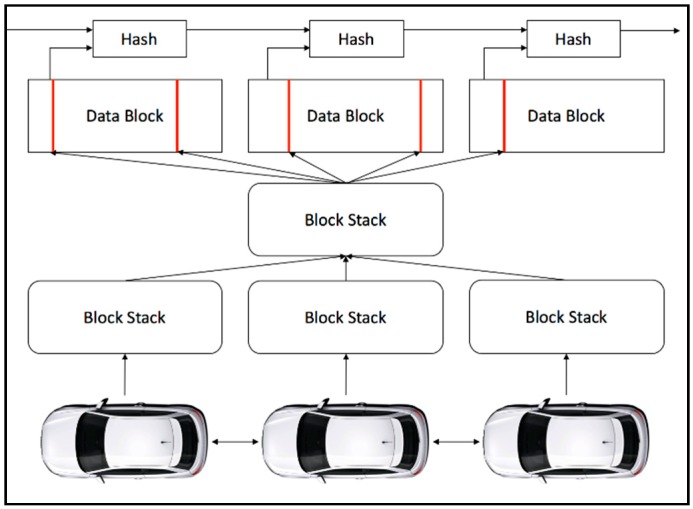
Generation of blockchain from unconfirmed event messages.

A newly minted block is mined by all the miners in the independent blockchain, which will then be sent to the local blockchain network. The blockchain is the medium to measure the trustworthiness within the local blockchain network or the country itself, as it acts as the global ground truth for the vehicular nodes. To be precise, any automobiles in the network have the potential to query the trust level of a vehicular node at any time during an event. The unconfirmed event messages are sorted and generate a new block due to aggregation from the message pool. Figure 3 shows the blocks with hashes that are chained in a sequential order to build a blockchain. The new blocks are broadcasted after the generation of it, where all the automobiles in the network verify and update the chain of the blockchain. 

### 4.1. Design of Proposed Blockchain Architecture

**Figure 4:**
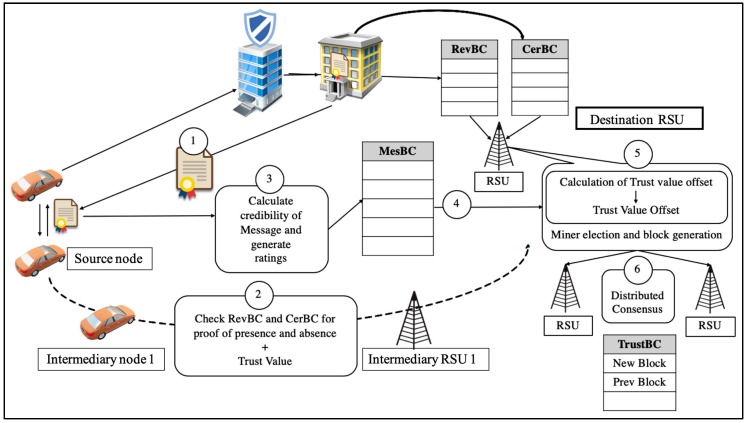
Proposed blockchain architecture in VANET.

Blockchain technology is implemented at the MAC layer (layer 2) in the proposed solution to ensure secure and trustworthy communication across the network. Furthermore, the proposed solution also integrates a signatureless public key infrastructure to preserve the privacy of the users in the network. Hence, this study endeavors to improve the security and privacy of users and messages communicated in VANET by tackling malicious attacks that are intended to bring catastrophic impacts on the network and other users. The continuous expansion of data in VANET through the implementation of the above technique has the tendency to affect the lightweight feature of the system over time [36]. However, treating the data in VANET as big data can help solve the complexity of the system. Data aggregation, storage, transmission, and computation are the four essential components that need to be well established in order to preserve the lightweightness of the proposed solution. To support big data, 5G technologies will be sufficient to support the VANET service with the implementation of the proposed architecture. Key performance indicators state that 5G networks are capable of offering a 10 Gb/s data rate, with less than 1 millisecond end-to-end latency [37]. Moreover, the three categories of use cases defined in 5G, enhanced mobile broadband (eMBB), ultra-reliable and low-latency communication (URLLC), and massive machine-type communication (mMTC), are well-characterized key innovations that can give ensured execution to VANET’s big data gathering and transmission tasks [38]. Figure 4 shows the system design of the proposed solution, in which a communication occurs between two vehicles. In this study, four different blockchains were considered for the system design—the certificate blockchain (CertBC), revocation blockchain (RevBC), message blockchain (MesBC), and trust blockchain (TrustBC). All of these blockchains are administered by government agencies, such as the law enforcement agency (LEA) and certification authority (CA), because the proposed solution may require legal actions and legal usage of data for investigation. The standard submission regulation is to be set by the government agencies and it is to be adhered by the respective automakers registered in the country for the participation of their vehicles in the VANET. 

**Figure 5:**
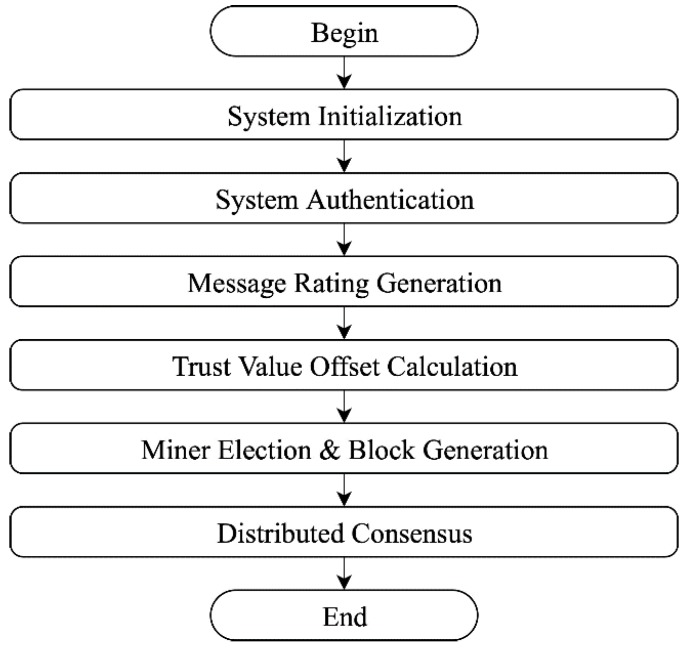
Flowchart of proposed solution.

There are six phases in the proposed solution: system initialization, system authentication, message rating generation, trust value offset calculation, miner election and block generation, and distributed consensus, as shown in Figure 5. The first phase of the proposed solution begins with a system initialization. This phase is responsible for validating the identity of the nodes and issuing a certificate to them when nodes move into a network. The subsequent phase is system authentication, which acts as a security layer to authenticate nodes before the nodes can begin communicating with each other in the network. Next, message rating generation is concerned with providing a rating on the messages sent by the communicating nodes to ensure their trustworthiness. Following this, the fourth phase is trust value offset calculation, which is required to calculate the trustworthiness of each node in the network. After that, the system conducts a miner election and block generation, which implements the blockchain technology for an efficient tracking of the nodes in the system. The last phase of the proposed solution is the distribution of consensus, which acts as a ledger that is spread around the network.

#### 4.1.1. System Initialization

**Figure 6:**
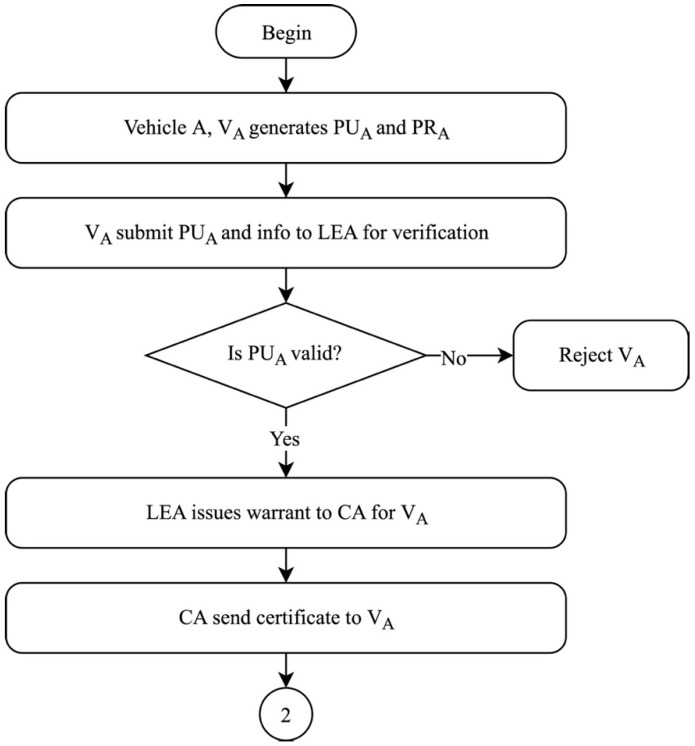
System initialization of proposed solution.

A node enters VANET, known as Vehicle A, V_A_. As V_A_ enters the network, the first measure of initialization is the generation of its public and private keys, PU_A_ and PR_A_, respectively. The generated PU_A_, along with a set of private information of V_A_, is then submitted to LEA for verification. If the verification of the materials by LEA is valid, LEA then issues a warrant to the CA for the certification of V_A_. When CA receives the warrant from LEA, it sends a valid certificate to V_A_, known as C_A_. However, if the materials are not verified by LEA as valid, then LEA rejects V_A_. Once V_A_ is verified and receives C_A_, V_A_ enters phase 2, system authentication, as shown in Figure 6.

#### 4.1.2. System Authentication

**Figure 7:**
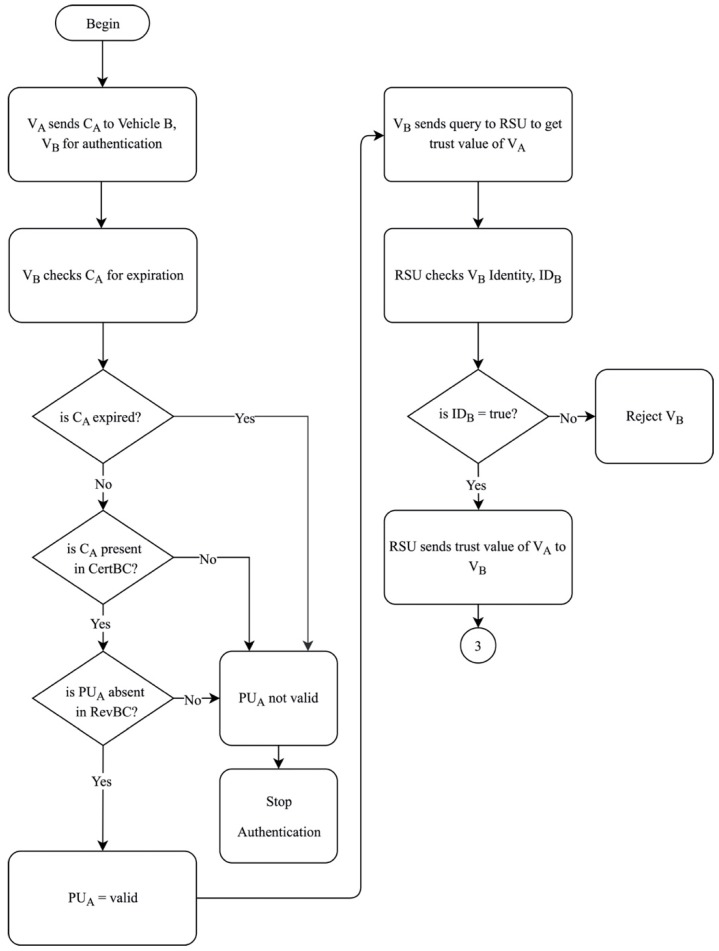
System authentication of the proposed solution.

The second phase is crucial for authenticating the vehicles in the network prior to communicating and exchanging data with each other. Assuming there is now another vehicle, Vehicle B, in the same network, known as V_B_, with which V_A_ wishes to communicate. This phase begins with V_A_ sending its C_A_ to V_B_ to authenticate the identity of V_A_. When V_B_ receives the certificate from V_A_, V_B_ checks whether the C_A_ delivered is valid as of the delivery date and time. The certificate would contain the public key and signature of V_A_ as well as the expiration date of C_A_. In order to authenticate V_A_, V_B_ checks the expiration of C_A_. If C_A_ has not expired, C_A_ is still valid. Then, V_B_ checks CertBC to see if C_A_ is present in the blockchain. If C_A_ is present in CertBC, then V_B_ proceeds to check RevBC to see if the PU_A_ is absent in the blockchain. If all these three conditions are fulfilled, only then will the communication between V_A_ and V_B_ take place. Failure of any of the three conditions mentioned would indicate that either the C_A_ has expired and is invalid or the PU_A_ is invalid. Hence, the authentication process will be halted immediately. When the identity of V_A_ is authenticated, V_B_ then sends a query to the nearest RSU to obtain the current trust value of V_A_. Initially, RSU checks the identity of V_B_ using the ID of V_B_, ID_B_. If the ID_B_ is valid, RSU then obtains the trust value of V_A_ from TrustBC and sends the trust value to V_B_. However, V_B_ is rejected if the ID_B_ is found to be not true. Once the trust value is calculated, V_B_ will receive the details of V_A_ and proceeds to the third phase, as shown Figure 7.

#### 4.1.3. Message Rating Generation

**Figure 8:**
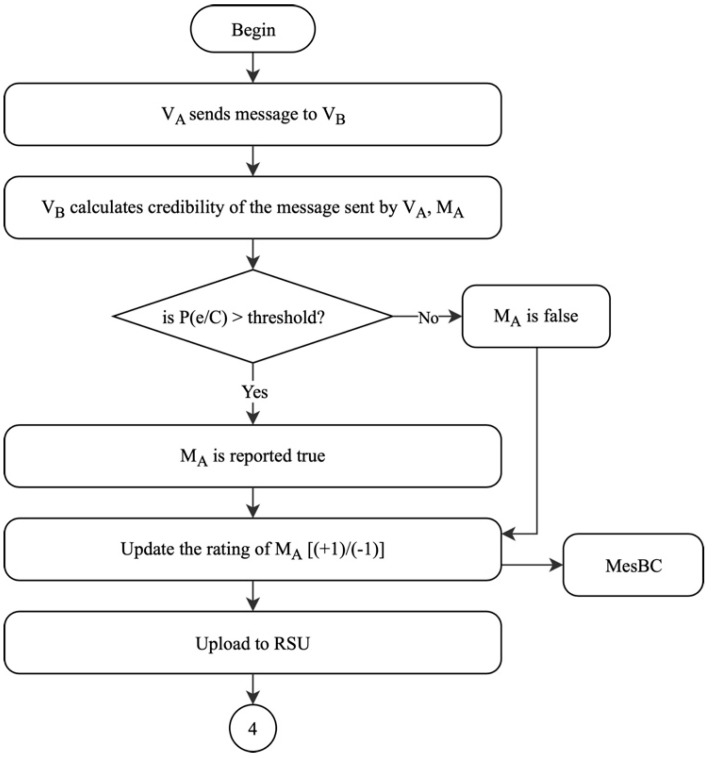
Message rating generation of the proposed solution.

Once the identities of the communicating vehicles are authenticated, the vehicles can now exchange data between them. Message rating generation is the third phase, which will officially initiate communication between the nodes. When V_A_ sends a message of a particular event, M_A,_ to V_B,_ V_B_ calculates the credibility of M_A_ to determine the trustworthiness of the message. Messages that report similar incidents or events are recorded in the MesBC. Therefore, for every vehicle that reports similar events occurrence in the network, the trust of the events is calculated and stored in a trust set. Next, the probability of such events, known as P(e/C), occurring is calculated using the trust set. If the calculated P(e/C) is greater than the existing threshold value, then M_A_ will be reported as true. However, if P(e/C) does not exceed the threshold, then M_A_ will be reported as a false report. When M_A_ is true, V_B_ generates a positive rating on the message received from V_A_. If otherwise, V_B_ generates a negative rating on that message. Then, V_B_ stores the messages received in the MesBC blockchain. The MesBC, along with the ratings given on each message, will be then uploaded periodically to a nearby RSU by V_B_ before proceeding to the next phase, as shown in Figure 8. The message ratings will indirectly reflect the trustworthiness of the source vehicle, which in this case is V_A_. Therefore, if a vehicle has more positive ratings, the vehicle can be identified as a trustworthy vehicle in the network. On the contrary, if a vehicle is found to have more negative ratings, the certificate and public key of the vehicle will be revoked by the LEA. Firstly, when the vehicle consistently receives negative ratings exceeding the threshold defined by the LEA, the vehicle will be temporarily blocked from sharing further information with other vehicles in VANET. Next, a direct report will be sent to the LEA for further action within 24 h. This will ensure that the revocation time is maintained at an acceptable range, which will allow the LEA to investigate the behavior of the vehicle. This algorithm, as shown in Algorithm 1, can mitigate false data injection attack, as the ratings will help users in the network to identify and differentiate between trustworthy vehicles and untrustworthy vehicles.

**Algorithm 1** Message rating generation**Require**: *M_j_*: Message group reporting event *e_j_* broadcasted by Vehicle *V_i_* (*i* = 1, 2, …*n*); C^j^_i_: credibility of *M_j_* as reported by *V_i_*; C*^j^* {}: credibility set for event *e_j_*; P(e/C): probability of event *e*; *Thr*: threshold of event probability; *R’_i_*: current rating of *V_i_* **Ensure**: *R_i_*: Updated rating of *V_i_*1:  **if** (*i* = 0) **then**2:    C^j^_i_ = 03:  **else for** each *V_i_*
**do**4:    calculate C^j^_i_5:        C*^j^* {}← C^j^_i_6:        **end for**7:    calculate P(e/C) using C*^j^* {}8:    **if** (P(e/C) > *Thr*) **then**9:    **for** each (*M_j_* = true) **do**10:       *R_i_* ← *R’_i_* + 111:         **end for**12:     **else for** each (*M_j_* = false) **do**13:       *R_i_* ← *R’_i_* − 114:         **end for**15:        **end if**16:     **end if**

#### 4.1.4. Trust Value Offset Calculation

**Figure 9:**
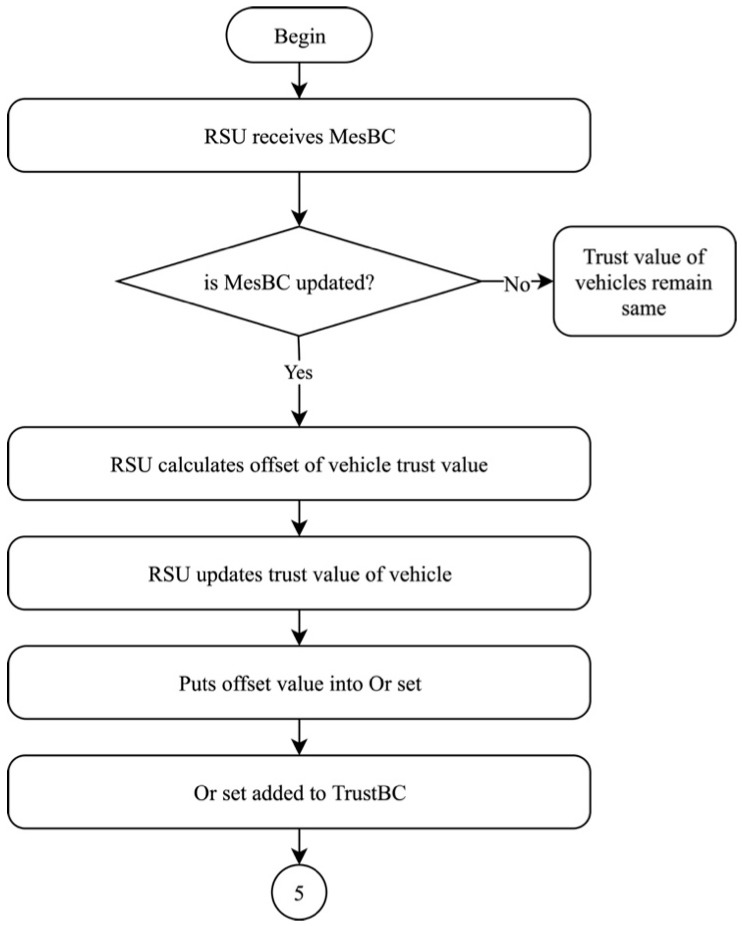
Trust value offset calculation of the proposed solution.

The fourth phase of the proposed solution begins as the RSU receives MesBC from V_B_, as illustrated in Figure 9. This phase calculates the trust value offset of each vehicle (in this case, V_A_) in the network. Once the RSU receives MesBC, it first checks if MesBC is updated from the previous uploads. If MesBC is not updated, the trust value of V_A_ remains the same. However, if MesBC is updated, RSU begins calculating the trust offset for V_A_. If V_A_ has not sent any message to V_B_ prior to the latest upload of MesBC, then the trust value of V_A_ remains the same as it was in the previous upload. If V_A_ did, indeed, send a message to V_B_, then RSU obtains the updated rating of V_A_. Then, RSU calculates the offset of vehicle trust value. Next, RSU updates the trust value of V_A_ in the network. RSU also updates the offset of trust value into a set of trust offset, known as O_r_, which is then uploaded into TrustBC, before moving to the following phase in the proposed solution. Algorithm 2 shows the trust value offset calculation for the proposed solution.

**Algorithm 2** Trust value offset calculation**Require**: *R_i_*: Updated rating of *V_i_*; *T’_i_*: current trust value of *V_i_*; *O_r_* {}: RSU *r* set of offsets **Ensure**: *T_i_*: Updated trust value of *V_i_*1:  **if** (*i* = 0) **then**
2:    *T_i_* ← *T’_i_* + 03:  **else for** each *V_i_*
**do**4:    **get**
*R_i_*5:        calculate offset6:        *T_i_* ← *T’_i_*7:        *O_r_* {} ← *T_i_*8:        **end for**

#### 4.1.5. Miner Election and Block Generation

**Figure 10:**
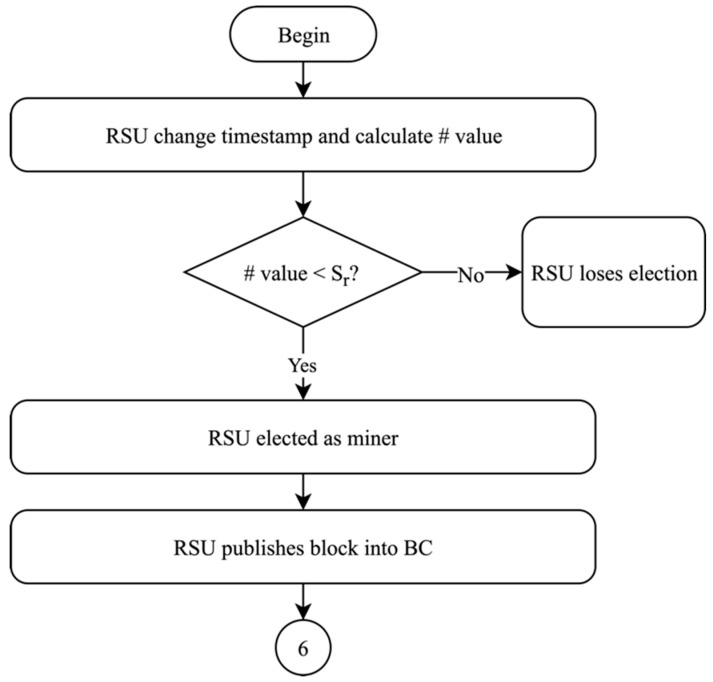
Miner election and block generation of the proposed solution.

Following trust value offset calculation in phase four, the proposed solution then moves into phase five, which is concerned with miner election and block generation. The fifth phase is the shortest of all the phases; however, it has the most significance to the efficiency of the proposed solution, as it implements blockchain technology in the network. Each RSU in the network registers its timestamp and calculates the hash value. If the calculated hash value of RSU_r_ is lower than the threshold S_r_, then RSU_r_ crosschecks its sum of absolute values of trust offset with the maximum sum of absolute values. If the sum of absolute values of RSU_r_ is lower than the maximum sum, then RSU_r_ is elected as the miner. If RSU_r_ fails any of these two conditions, then RSU_r_ loses the election. The election of miner RSU moves to the next RSU in the network and repeats the steps mentioned. When miner RSU is elected, the miner publishes its block into blockchain. The flow of the fifth phase is shown in Figure 10. A miner RSU is elected periodically in the network to manage the blockchain due to the decentralized structure of the blockchain technology. The election of miner RSU ensures the update of data in the blockchain in a timely manner.

**Figure 11:**
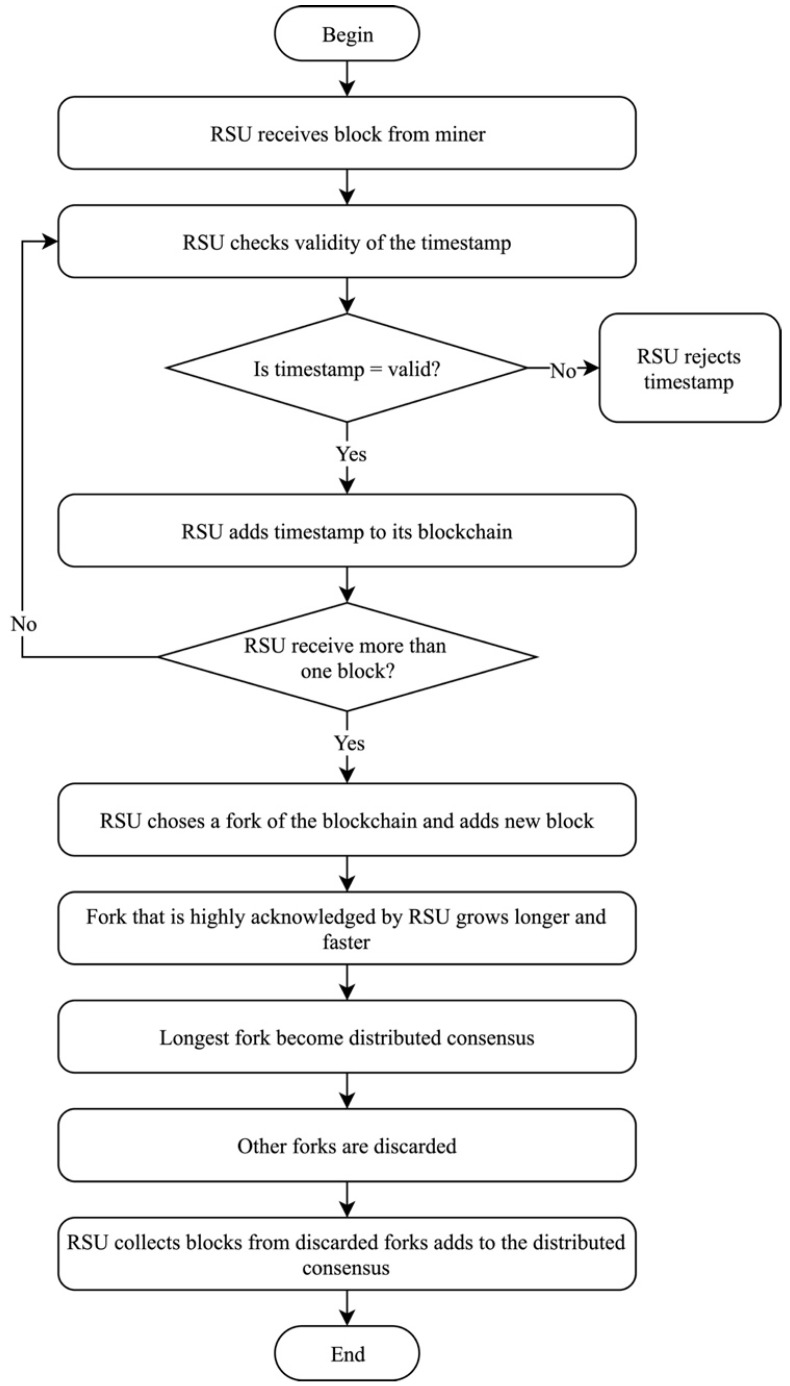
Distributed consensus of proposed solution.

When other forks are discarded, RSUs gather their respective blocks from the discarded forks and add them to the distributed consensus, as illustrated in Figure 11. The sixth phase ensures that all RSUs in the network have the same blockchain, which results in consistency of the data.

## 5. Simulation Tool and Set-Up

In this paper, network performance analysis was chosen to analyze the influence of the proposed work on the network with and without the presence of denial of service attack. The proposed work was compared with several existing protocols: authentication based on smart card (ASC); lightweight authentication and key agreement protocol (LAKAP); hybrid approach for privacy-preserving authentication scheme (HEPPA); efficient, scalable, and privacy-preserving authentication (ESPA); and secure privacy-preserving authentication with cuckoo filter (SPACF).

### 5.1. Selection of Simulation Tool

The selection of a network communication simulator is highly crucial when it comes to potentially transmitting several messages per second when simulating a VANET environment with a large number of vehicles. The first assumption about the automobiles in the network was that every vehicle is able to communicate with other entities either by V2V and V2I communications that can associate with the internet connectivity perfectly [39]. Every automobile was assumed to be equipped with three important components, called the OBUs, sensors, and GPS. A total of 10 RSUs were spread over 1 km radius [40]. The total number of RSUs that were malicious were considered to be less than the number of non-malicious RSUs that were placed alongside the roadway in a network. A genesis block was able to create and start a blockchain based on local events if it was an RSU that was legitimate and the certificate authority (CA) was a trusted entity. Besides this, the participating automobiles were assumed to be able to handle high computing power and have a high trust level which was also considered a complete vehicular node that could take part in the mining process. In addition, the malicious vehicles could not outrun the number of total vehicles in the network, since it had not much capacity to compromise more than one to the ratio of two. Another assumption taken into consideration was that a received signal strength indicator (RSSI) is included together with the critical event messages in a specific geographical location [41]. The unencrypted critical messages were also available to other neighboring automobiles in real time and the timers of all the nodes were synchronized. Lastly, a total of 15 event messages was required to confirm a new event that was reported through the critical messaging to testify an event to be truthful.

Veins was chosen as the simulation tool for this study due to the following features: online re-configuration and re-routing of vehicles in reaction to network simulator, fully-detailed models of IEEE 802.11p and IEEE 1609.4 dedicated short-range communications/wireless access in vehicular environments (DSRC/WAVE) network layers, supporting the realistic map and traffic scenario, user friendliness, and the ability to interconnect. The Veins hybrid simulator was chosen in order to achieve the bidirectional coupled simulation, with benefits from state-of-the-art simulation techniques of both the network simulation and the road traffic micro simulation domains. In Veins, objective modular network testbed in C++ (OMNeT++) works as the network simulator and simulation of urban mobility (SUMO), on the other hand, acts as the road traffic simulator. The traffic control interface (TraCI) integrated both SUMO and OMNeT++ to provide a transmission control protocol (TCP) connection between the simulators. A real time communication between the network simulation module and road traffic simulation module could be generated in Veins. Besides this, the network simulation module was able to influence the road traffic simulation module.

### 5.2. Simulation Environment Set-Up

The parameters and values set for the SUMO simulator were as shown in Table 2. The parameters set fixed were as follows: number of nodes, maximum vehicle speed, maximum acceleration, maximum deceleration, vehicle length, vehicle width, and driver imperfection. Table 3 shows the fixed variables set up for OMNeT++ simulator with a set of parameters as follows: Sim-time-limit, Mac.queuelength, Mac.maxTxAttempts, Mac.txpower, Mac.bitrate, Mac.contentionWindow, Mac.slotduration, Phy.sensitivity, and UpdateInterval.

## 6. Results and Discussion

This section presents and discusses the experimental analysis of the efficiency of the proposed trust-based communication algorithm in VANET through simulation. The performance of the algorithm for VANET was evaluated in terms packet delivery ratio, end-to-end delay, packet loss, and packet overhead using the selected Veins simulation tool. Each performance analysis was run 10 times in the simulator and then a statistical analysis was performed by averaging the values obtained to a mean value of the reading to be compared with the benchmark protocols, as well as the confidence interval (CI) of the results obtained for each evaluation metric, both with and without the presence of a denial of service attack, which was simulated using NETwork Attacks (NETA). 

### 6.1. Packet Delivery Ratio (PDR)

PDR refers to the ratio of the number of packets that were successfully received to the total number of packets sent in the network [42]. PDR was obtained by determining the ratio of total number of packets received, $P_{r}$, to the total number of packets sent, $P_{s}$, in the network, as shown in (1):

(1) $$PDR\;=\;\frac{P_{r}}{P_{s}}\;\times \;100\%.$$

**Figure 12:**
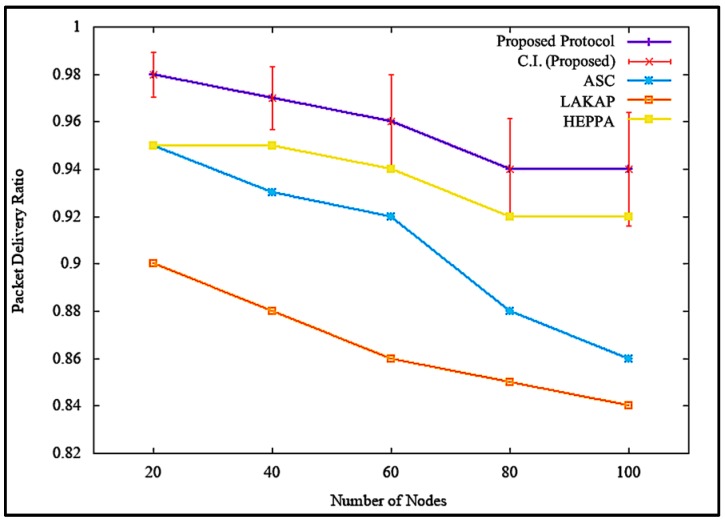
Packet delivery ratio (PDR) without denial of service attack.

Figure 12 illustrates the impacts of PDR when the attacker or malicious node was non-existent. Figure 12 shows that the proposed solution incurred higher PDR, with a difference of 8.0%, 10.0%, and 2.4% as opposed to ASC, LAKAP, and HEPPA, respectively. In terms of statistical analysis, the proposed solution resulted in an average PDR value of 0.94, standard deviation of 0.033483, and a confidence interval of 0.023952 with 100 nodes in the network. During execution, all solutions showed a slump in PDR value as the number of nodes increased. This is due to the communication channel becoming congested with the nodes trying to transmit data across the network, therefore resulting in packet drops.

**Figure 13:**
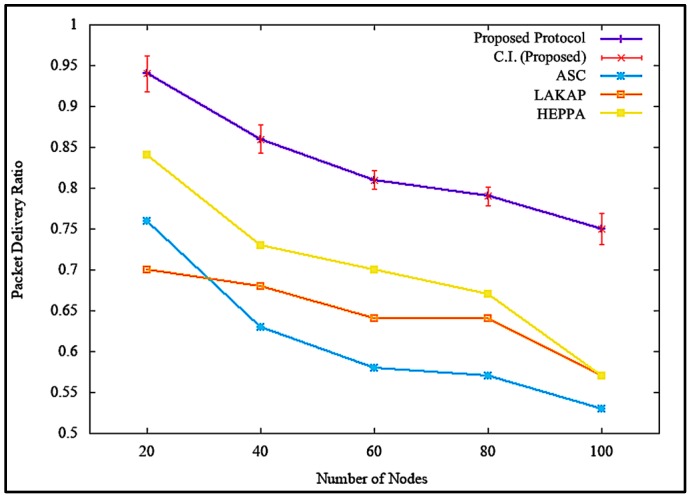
PDR with denial of service attack.

However, the PDR of the proposed solution was higher with a much steadier decrease as the number of nodes were increased because the proposed solution involved a lower intense computational operation in order to execute the algorithm. This caused a decrease in the delay of packet transmission, resulting in higher PDR. Similarly, it can be seen from Figure 13 that the proposed solution maintained PDR at a tolerable level with a difference of 21.5%, 17.7%, and 17.5% as opposed to ASC, LAKAP, and HEPPA, respectively, which showed rapid slump when the number of nodes was increased from 80 to 100 when a denial of service (DoS) attack was executed in the network. In terms of statistical analysis, the proposed solution resulted in an average PDR of 0.75, standard deviation of 0.027264, and a confidence interval of 0.019504 with 100 nodes in the network. Based on Figure 13, the PDRs of the benchmark protocols were much lower than the proposed solution, because the algorithms were not able to properly protect the network from the DoS attack, resulting in a higher loss of packet.

### 6.2. End-to-End Delay

End-to-end delay is defined as the time taken for a packet to arrive at the destination from the source [43,44]. PDR was significantly impacted by the end-to-end delay experienced by the network. End-to-end delay was calculated by considering the difference between the arrival time of a packet at its destination vehicle and the sent time of a packet at its source vehicle. Equation (2) shows the formula for end-to-end delay calculation, where EED denotes end-to-end delay, $T_{A}$ denotes arrival time of a packet, and $T_{S}$ denotes sent time of a packet:

(2) $$EED\;=\;\sum T_{A}-\;T_{S}.$$

**Figure 14:**
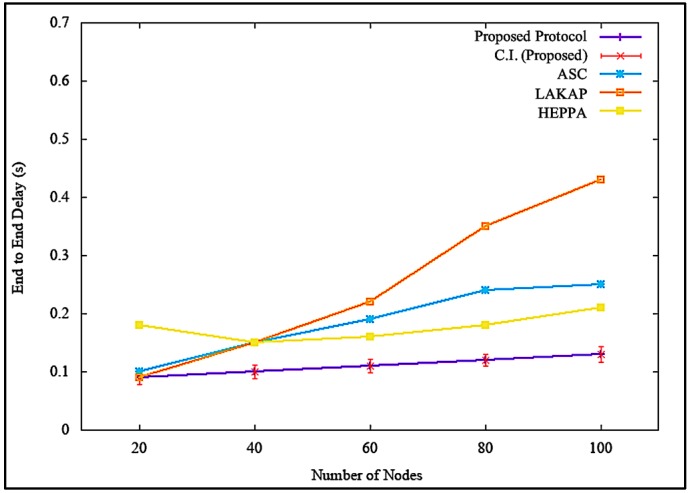
End-to-end delay without denial of service attack.

Figure 14 shows the simulation results of end-to-end delay incurred by the network when there was no attack executed. Figure 14 shows that the proposed solution incurred a steady, lower end-to-end delay with a difference of 0.12 s, 0.30 s, and 0.08 s as compared to ASC, LAKAP, and HEPPA, respectively. LAKAP showed a significant hike in end-to-end delay when the number of nodes as increased from 60 to 80 compared to the rest of the three solutions. In terms of statistical analysis, the proposed solution resulted in an average end-to-end delay of 0.13 s, standard deviation of 0.018738, and a confidence interval of 0.013404 with 100 nodes in the network. Overall, the proposed solution incurred a lower and steadier end-to-end delay because of the lightweight hashing technique with sponge construction, which relies only on single permutation. This implies that the data packets were processed in a shorter time. 

**Figure 15:**
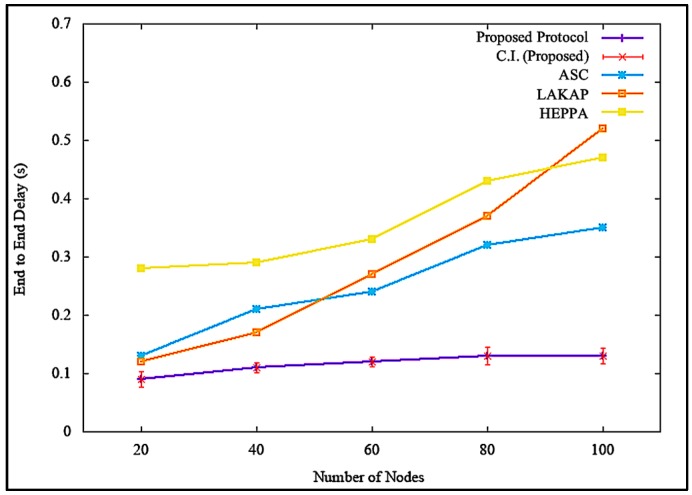
End-to-end delay with denial of service attack.

Similarly, it can be seen from Figure 15 that the proposed solution maintained a steady yet minor end-to-end delay time, with a difference of 0.22 s, 0.39 s, and 0.34 s as opposed to ASC, LAKAP, and HEPPA, respectively, when a denial of service attack was executed in the network. When a statistical analysis was performed, the proposed solution results maintained an average end-to-end delay of 0.13 s but with a slightly higher but far more tolerable standard deviation of 0.017638 and a confidence interval of 0.012618. The values were achievable due to the lightweight blockchain format in the proposed solution, linked via previous hash of message blocks, which also stands as a challenge for attackers to execute denial of service attack and modify data packets.

### 6.3. Packet Loss

Packet loss ratio is defined as the proportion of packets dropped against the total number of packets sent in the network. Packet loss ratio, PLR, was obtained by determining the ratio of total number of packets lost, $P_{l}$, to the total number of packets sent, $P_{s}$, in the network, as shown in (3):

(3) $$PLR\;=\;\frac{P_{l}}{P_{s}}\;\times \;100\%.$$

**Figure 16:**
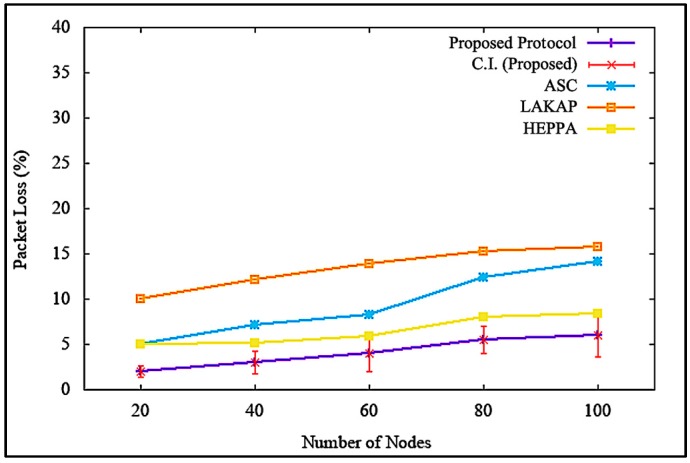
Packet loss ratio without denial of service attack.

Figure 16 illustrates the impacts on packet loss when the attacker or malicious node was non-existent. It shows that the proposed solution incurred lower packet loss with a difference of 8.0%, 10.0%, and 2.4% as opposed to ASC, LAKAP, and HEPPA, respectively. As for the average reading at 100 nodes, the value was 6%, which was the lowest among the three benchmarks, with a standard deviation of 3.299832 and a confidence interval of 2.360557. The packet loss of the proposed solution was lower because the proposed solution involved a lower computationally intense operation in order to execute the algorithm. This caused a decrease in the delay of packet transmission, resulting in lower packet loss. 

**Figure 17:**
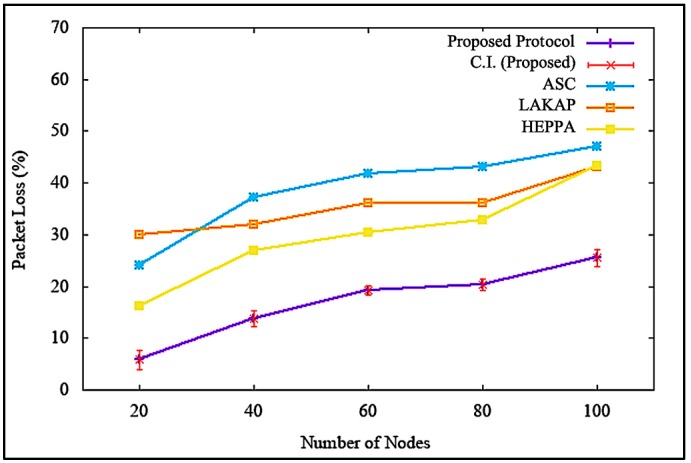
Packet loss ratio with denial of service attack.

Similarly, it can be seen from Figure 17 that the proposed solution maintained packet loss at a tolerable level, with a difference of 21.5%, 17.7%, and 17.5% as opposed to ASC, LAKAP, and HEPPA, respectively, when a DoS attack was executed in the network. In terms of statistical analysis, the average packet loss was 25.5%, with a standard deviation of 2.321398 and a confidence interval of 1.660628. The packet loss of the benchmark protocols was much higher than the proposed solution because the algorithms were not able to protect network from the DoS attack as efficiently as the proposed solution since the beginning, resulting in a higher number of packet-drops, as shown in Figure 17.

### 6.4. Transmission Overhead

Transmission overhead refers to the number of overhead bytes divided by the total number of bytes in a packet transmission. Longer data packets are heavier, as they have to carry more data in a packet, resulting in higher transmission overhead.

(4) $$O_{T}=\;\frac{bytes_{overhead}}{bytes_{total}}.$$

**Figure 18:**
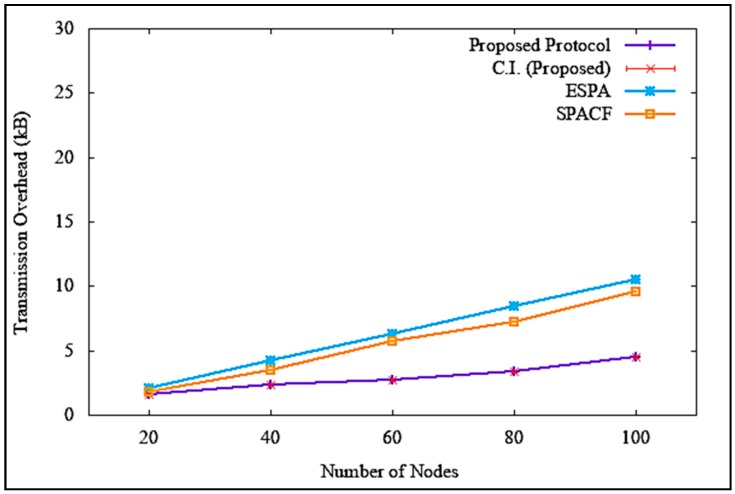
Transmission overhead without denial of service attack.

**Figure 19:**
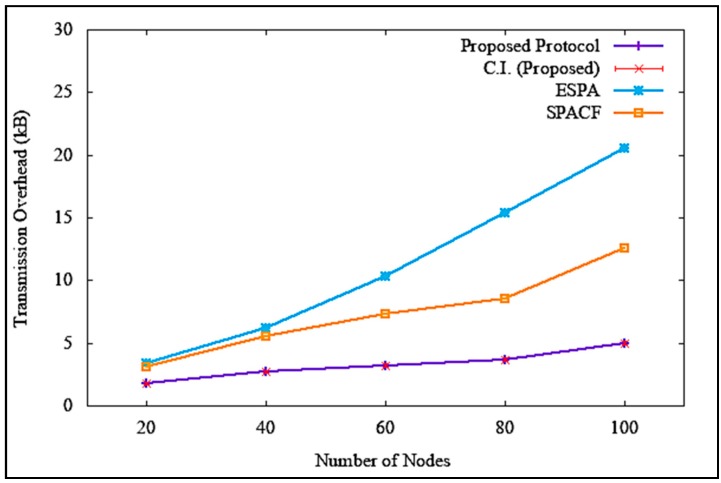
Transmission overhead with denial of service attack.

Equation (4) shows the formula of transmission overhead calculation, where $O_{T}$ denotes transmission overhead, $bytes_{overhead}$ denotes overhead bytes, and $bytes_{total}$ denotes the total number of bytes in a transmission. The results of transmission overhead when the attacker or malicious node was non-existent are illustrated in Figure 18, which clearly shows that the transmission overhead increased with an increase in the number of nodes. However, it is clear in the figure that the proposed solution incurred a much lower transmission overhead, with a difference of 6 kB and 5.1 kB as opposed to ESPA and SPACF, respectively. At 100 nodes, the average transmission overhead was 4.5 kB, with a standard deviation of 0.149443 and a confidence interval of 0.106905. Similarly, when there was an attack, as shown in Figure 19, the proposed solution incurred a steady increase in transmission overhead as compared to ESPA and SPACF, suffering a drastic increase in transmission overhead, with a difference of 15.5 kB and 7.6 kB, respectively. At 100 nodes, the average transmission overhead was 5.0 kB, with a standard deviation of 0.207913 and a confidence interval of 0.148722. The proposed solution incurred a lower transmission overhead compared to the benchmark protocols because of its implementation of a signatureless public key infrastructure authentication technique to maintain a lighter and shorter packet header.

### 6.5. Computational Cost

**Figure 20:**
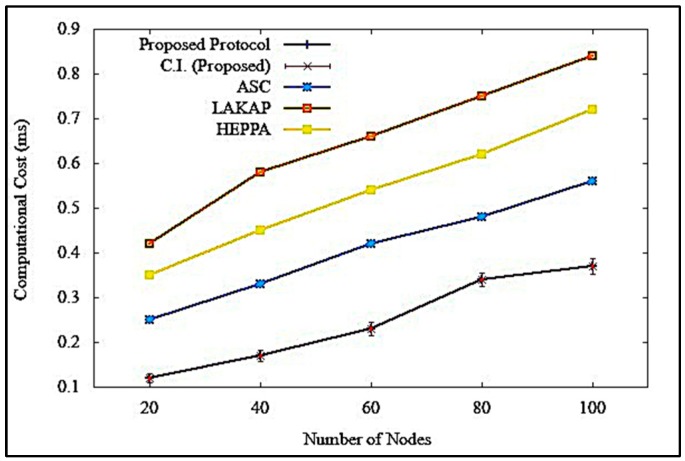
Computational cost of the proposed solution and existing solutions.

Figure 20 illustrates the impacts of computational cost when the proposed solution was executed. Figure 20 shows that the proposed solution incurred lower computational cost, with a difference of 0.19 ms, 0.47 ms, and 0.35 ms as compared to ASC, LAKAP, and HEPPA, respectively. In terms of statistical analysis, the proposed solution resulted in an average computational cost of 0.37 ms, standard deviation of 0.023483, and a confidence interval of 0.017952 with 100 nodes in the network. During execution, all solutions showed a jump in computational cost value as the number of nodes increased from 60 to 80. This was due to the adaption of big data technology in the network that had a lower latency. The computational cost of the proposed solution was lower, as the number of nodes were increased because 5G technologies can accommodate huge concurrent connectivity and provide reliable data transmission to facilitate big data gathering services. This caused a decrease in the complexity of computational operation, resulting in a much more efficient VANET protocol.

### 6.6. Security Analysis

Compared to the benchmark protocols, the proposed solution performed better in terms of security. The uniqueness of blockchain technology is that once the information is recoded and endorsed in the blockchain, then it is impossible to overwrite, modify, or even delete it from the network. Additionally, no information can be added randomly, which is one unique and important feature of blockchain. Secondly, any of the contents of the blockchain included by the nodes in the system were distributed among the nodes in the network to synchronize and validate without the help of a central control. A single point of failure was not possible with this system and it was much more secure as it provided a trustworthy environment. Moreover, a blockchain environment comes with a privacy protection to the users, whereby a user can be a part of the network without revealing their identity. For example, the user information is kept anonymous while sharing block details to the nodes in the network. In other words, personal information is private and confidential, plus secure. The reason for accurate, reliable, consistent, timely, and widely accessible data in the blockchain system is due to the decentralization of the network. The hash function processes input messages of various sizes and creates output messages of rigid size. The computation for this process includes the message authentication code (MAC), data integrity, and digital signatures. The hash function becomes free from collision when it can project data of random length to strings of rigid length. For the proposed solution’s hashing function, sponge construction was adopted, as it features a very lightweight design. The data could have been attacked by malicious attackers and yet did not fall into any single point failures. This is also the reason why the data were accurate and reliable. Any transactions or events that take place in the system will be updated from time to time in the blockchain. So, it is hardly possible to doubt the transparency of the transactions in the network. A consensus mechanism in a blockchain technology is a fault-tolerant mechanism that is advantageous to a single state of network among the distributed multi-node system in achieving the required agreement. The agreement is a list of rules and regulations for all the different participating nodes which will be helpful in deciding their contributions eventually. Some criteria are important in a decentralized blockchain network, such as the security, efficiency, reliability, and real-time information sharing of the publicly shared database to agree upon the participation of a node of a particular consensus and to prove the trustworthiness of the transactions within the network. 

From the above discussion it can be analyzed that our proposed mechanism can ensure confidentiality, integrity, availability, and non-repudiation. In terms of confidentiality, the use of elliptic curve cryptography encryption for all V2V and V2I communications is capable of mitigating data modification attack, impersonation attack. From an integrity point of view, each communication includes a hash and a timestamp of all other fields contained in the communication. Each block is linked with the previous hash; hence all communications are chained together, making it impossible for Sybil and replay attacks to occur. Last but not least, based on the experiment done, denial of service attacks does not seem to affect VANET, as the trust management algorithm only allows legitimate nodes to participate in the network and isolate possible malicious nodes before they cause irregularities in the network. Moreover, the decentralized nature of the blockchain architecture makes the VANET prone to severe downtime in the case of other external issues, securing the network availability at all times. In our future work, formal methods will be used to conduct extensive security analysis.

### 6.7. Secure Routing Mechanism

Although secure routing mechanism was not the specific focus of this research work, it is worthful evaluating the routing-based network performance once our proposed scheme was utilized. During vehicular network communication, data packets move through intermediate nodes from source to destination. Due to highly dynamic mobility and frequent change in topologies, the chances of link breakage are substantially high. Thus, it is not advisable to maintain per-destination routing information. In this research, we investigated the geographical routing protocol greedy perimeter stateless routing (GPSR), which only keeps local information of the neighbor. However, GPSR is proven to be exposed to various insider attacks [45,46]. Later, secure-geographical routing protocol greedy perimeter stateless routing (S-GPSR) was proposed to address the security concerns of the baseline protocol. Thus, we adopted the S-GPSR routing protocol to execute our proposed scheme. 

**Figure 21:**
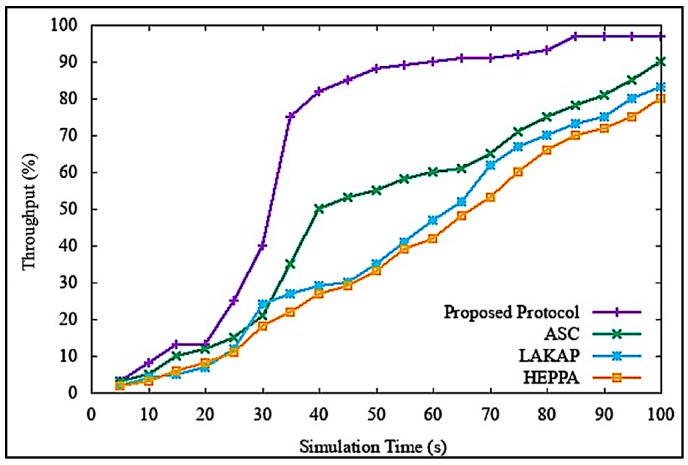
Percentage of throughput with the secure-geographical routing protocol greedy perimeter stateless routing (S-GPSR) protocol.

In this protocol, during packet forwarding to the destination, each node must scan its neighborhood routing table to obtain the next hop information, thus it selects the node with high trust value, also known as the highly trusted route, rather than the default minimum distance. S-GPSR uses the trust update interval (TUI) model to decide the duration of time waited before calculating the trust values to each node. Each node in S-GPSR performs two functions, forwarding the packets and checking the integrity of the packets. The trust values of the participating nodes increase if the nodes successfully forward the packets in an appropriate manner and succeed in the integrity check. In case the integrity check fails and packets are not broadcasted in an appropriate way, the trust values decrease and the nodes are considered as malicious nodes. Figure 21 demonstrates the influence of overall network performance in terms of throughput once denial of service attack is induced in networks. When the proposed scheme was executed by utilizing the S-GPSR routing protocol, it showed a greater throughput of up to 50% as compared to other baseline security protocols. This was due to our proposed scheme utilizing its self-generated blockchain-based security mechanism, where evaluation of integrity and trust are embedded within each participating device. Thus, other protocols required some additional processing delay to implement the security mechanism. This delay affected the packet deadline, which led to the data packets missing the end-to-end deadline. Furthermore, an in-depth study will be carried out in future work.

## 7. Conclusions

VANETs have received an enormous amount of attention from both researchers and the vehicular industry due to their potential in delivering information to provide safety and infotainment messages to drivers and passengers. Unfortunately, trust management for vehicles is still an open issue in VANETs. Therefore, this research proposed a secure trust-based blockchain architecture to effectively mitigate several network attacks while preserving the privacy and security of the users in VANET. The proposed solution was developed to mitigate networks attacks, such as message fabrication, impersonation, DoS attacks, and Sybil attacks, while maintaining the privacy of the users in VANET. The blockchain technology in the proposed solution uses timestamps and hashing techniques to maintain the freshness of the messages delivered. These techniques minimize message fabrication or modification attacks, as the timestamps record the time a message is delivered, while hashing secures the message against tampering by malicious nodes. Furthermore, the proposed solution also uses a message rating and credibility approach via the blockchain technology. The message rating and credibility approach ensures trust management among vehicles during information exchange in VANET. Any vehicle that communicates fake messages to other vehicles in the network will be rated with low values, decreasing its credibility. Vehicles with a lower trust value than the threshold value will be rejected from the network and their vehicle certificates will be revoked. The performance of the proposed solution was evaluated via simulation using the Veins simulation tool under two settings, which were without denial of service attacks and with denial of service attacks. From the simulation, the proposed solution was found to perform better than the benchmark algorithms in terms of the PDR against increasing number of nodes in the network. The simulations showed that the proposed solution experienced up to 98% of PDR when there were no attacks launched in the network, while during attack, the proposed solution incurred up to 94% of PDR. Interestingly, the proposed solution experienced a similar delay of 0.130 s over increasing number of vehicles in the network, with and without network attacks. Despite the improved performance of the proposed solution, this study was still bound to several research limitations. First, the proposed solution was only implemented and evaluated on one component of the intelligent transportation system—vehicular ad hoc network (ITS—VANET). Future works should include deployment of the proposed solution in autonomous vehicles and deployment in a multi-junction road network. Cooperative-ITS (C-ITS) is another component of ITS that supports connectivity [47] and cooperative awareness of road users, which can be achieved with regular exchange of safety information among users [48]. In the future, C-ITS will be a possible application to be integrated with the proposed solution and to investigate the performance of the proposed solution in enabling cooperative awareness in VANET.

## Figures and Tables

**Table 1:** List of notations and description used.

Notation	Description
V*_i_*	Vehicle *i*
PU*_i_*	Public Key of V*_i_*
PR*_i_*	Private Key of V*_i_*
C*_i_*	Certificate of V*_i_*
CertBC	Certificate Blockchain
RevBC	Revocation Blockchain
ID*_i_*	Identity of V*_i_*
M*_i_*	Message *i* from Vehicle *i*
TrustBC	Trust Blockchain
P(*e*/c)	Probability of Event *e*
MesBC	Message Blockchain
O*_r_*	Set of Offsets of Road Side Unit *r*
S*_R_*	Hash Threshold of RSU *r*
RSSI*_i_*	Relative Signal Strength Indicator Value of Vehicle *i*
t*_i_*	Timestamp of Vehicle *i*
h*_n_*	Hash Value of block

**Table 2:** Parameters set for the SUMO simulation.

Parameter	Values
Number of Nodes	100
Maximum Vehicle Speed	33 m/s
Maximum Acceleration	2.6 m/s^2^
Maximum Deceleration	4.5 m/s^2^
Vehicle Length	5 m
Vehicle Width	3.5 m
Number of RSUs	10
RSU Coverage Driver Imperfection	1 km 0.5

**Table 3:** Parameters set for the OMNeT++ simulation.

Parameter	Values
Sim-Time-Limit	6000 s
Mac.queuelength	5
Mac.maxTxAttempts	14
Mac.bitrate	11 Mbps
Mac.txpower	100 mW
Mac.contentionWindow	20
Mac.slotduration	0.04 s
Phy. Sensitivity	−80 dBm
UpdateInterval	0.1 s
